# Tumor Necrosis Factor Receptor-1 (p55) Deficiency Attenuates Tumor Growth and Intratumoral Angiogenesis and Stimulates CD8^+^ T Cell Function in Melanoma

**DOI:** 10.3390/cells9112469

**Published:** 2020-11-13

**Authors:** Yamila I. Rodriguez, Ludmila E. Campos, Melina G. Castro, Nadia Bannoud, Ada G. Blidner, Verónica P. Filippa, Diego O. Croci, Gabriel A. Rabinovich, Sergio E. Alvarez

**Affiliations:** 1 Instituto Multidisciplinario de Investigaciones Biológicas San Luis (IMIBIO-SL), Consejo Nacional de Investigaciones Científicas y Técnicas (CONICET) and Universidad Nacional de San Luis (UNSL), San Luis D5700, Argentina; yamilarodriguezmza@gmail.com (Y.I.R.); ludmilacampos86@gmail.com (L.E.C.); melinacastro6@gmail.com (M.G.C.); vpfilipp@gmail.com (V.P.F.); 2 Laboratorio de Inmunopatología, Facultad de Ciencias Exactas y Naturales, Instituto de Histología y Embriología de Mendoza (IHEM), Universidad Nacional de Cuyo, Consejo Nacional de Investigaciones Científicas y Técnicas (CONICET), Mendoza C5500, Argentina; nadiabannoud@gmail.com (N.B.); dcrocirusso@gmail.com (D.O.C.); 3 Laboratorio de Inmunopatología, Instituto de Biología y Medicina Experimental (IBYME), Consejo Nacional de Investigaciones Científicas y Técnicas (CONICET), Buenos Aires C1428, Argentina; adablidner@gmail.com (A.G.B.); gabyrabi@gmail.com (G.A.R.); 4 Departamento de Química Biológica, Facultad de Ciencias Exactas y Naturales, Universidad de Buenos Aires, Buenos Aires C1428, Argentina

**Keywords:** tumor necrosis factor receptor, B16.F1 melanoma, angiogenesis, immune microenvironment

## Abstract

The role of tumor necrosis factor-α (TNF-α) in shaping the tumor microenvironment is ambiguous. Consistent with its uncertain role in melanoma, TNF-α plays a dual role, either acting as a cytotoxic cytokine or favoring a tumorigenic inflammatory microenvironment. TNF-α signals via two cognate receptors, namely TNFR1 (p55) and TNFR2 (p75), which mediate divergent biological activities. Here, we analyzed the impact of TNFR1 deficiency in tumor progression in the B16.F1 melanoma model. Tumors developed in mice lacking TNFR1 (TNFR1 knock-out; KO) were smaller and displayed lower proliferation compared to their wild type (WT) counterpart. Moreover, TNFR1 KO mice showed reduced tumor angiogenesis. Although no evidence of spontaneous metastases was observed, conditioned media obtained from TNFR1 KO tumors increased tumor cell migration. Whereas the analysis of tumor-associated immune cell infiltrates showed similar frequency of total and M2-polarized tumor-associated macrophages (TAMs), the percentage of CD8^+^ T cells was augmented in TNFR1 KO tumors. Indeed, functional ex vivo assays demonstrated that CD8^+^ T cells obtained from TNFR1KO mice displayed an increased cytotoxic function. Thus, lack of TNFR1 attenuates melanoma growth by modulating tumor cell proliferation, migration, angiogenesis and CD8^+^ T cell accumulation and activation, suggesting that interruption of TNF-TNFR1 signaling may contribute to control tumor burden.

## 1. Introduction

Tumor necrosis factor alpha (TNF-α) is a pro-inflammatory cytokine initially identified because of its capacity to induce hemorrhagic necrosis in experimental tumors [1]. This effect caused high expectations regarding its possible use as an anti-tumor agent. However, it is clear that this cytokine also displays pro-tumoral functions, mainly through its ability to promote and sustain chronic inflammatory processes [2,3]. TNF-α is produced by many different cell types, including macrophages, T lymphocytes as well as natural killer (NK), stromal and tumor cells [2,4]. This cytokine signals through binding to two membrane receptors: receptor 1 (p55/TNFR1), found in almost all cell types and receptor 2 (p75/TNFR2) whose expression is limited to lymphoid cells, endothelial cells, neurons and human mesenchymal cells [5]. Signaling via TNFR1 induces apoptosis through the death domain located at the receptor cytoplasmic region or supports cell survival through activation of the nuclear factor (NF)-κB transcription factor [5]. On the other hand, TNFR2 induces tumor progression by stimulating NF-κB, ERK1/2 and AKT signaling [6].

The paradoxical role of TNF-α and its receptors either as promoters or inhibitors of cancer progression has been widely studied [2,3,7,8,9]. For example, in DMBA (7,12-dimethyl-benz(a)anthracene)-induced skin carcinogenesis, increased tumor progression correlated with elevated TNF-α levels [10]. In fact, genetic deletion of either TNFR1 or TNFR2 reduces mice susceptibility to chemically-induced skin carcinogenesis [11]. In ovarian cancer, TNFR1 enhances IL17^+^CD4^+^ T cell-dependent myeloid cell recruitment to tumor parenchyma leading to increased tumor growth [12]. Conversely, TNFR1 knock-out (KO) mice were more susceptible to inflammation-induced colon carcinogenesis [13]. Indeed, increased intestinal inflammation was also observed in mice lacking TNF [14]. Particularly, in melanoma, tumor growth was impaired in TNF KO and TNFR1 KO but not in TNFR2 KO mice injected with B16K1 cells, a genetically-modified cell line derived from B16F10 cells, which stably express the MHC-I molecule H-2Kb [15]. In contrast to wild type (WT) mice, TNFR1-deficient mice treated with anti-PD-1 blocking antibodies completely rejected growth of melanoma cells [16].

Interestingly, TNF-α also controls gene expression of tumor cells as well as other components of the tumor microenvironment, such as fibroblasts, myofibroblasts, adipocytes and immune cells. This observation is consistent with the broad regulatory function of TNF-α in the tumor microenvironment and illustrates the dual role of this molecule, acting either as a cytotoxic cytokine or as a tumor promoter that fosters an immunosuppressive microenvironment, controls tumor proliferation, angiogenesis, progression and metastasis [2,7]. Indeed, it has been shown that the immunosuppressive role of TNF-α could be related to activation of regulatory T cells (Tregs) or induction of T cell exhaustion [7]. Given the therapeutic value of monoclonal antibodies targeting the TNF-TNFR axis (infliximab and adalimumab) and a fusion protein (etanercept) that antagonizes TNF-α function in autoimmune inflammatory diseases, it has been proposed that modulating TNF-TNFR signaling, either with agonistic or neutralizing antibodies, may contribute to control tumor growth [17].

Melanoma, the deadliest form of skin cancer, arises from melanocytes. Melanocytes are characterized by the production of melanin, a pigment that protects normal melanocytes from ultraviolet radiation but also decreases the effectiveness of chemo- and radiotherapy [18]. Certainly, patients with pigmented melanoma have reduced overall survival and disease free survival [19] Although emerging therapies (particularly targeted therapies and checkpoint blockade immunotherapies) show an impressive success rate in melanoma patients, a great number of individuals display primary or acquired resistance to these treatments [20]. In this study, we explored the relevance of TNFR1 as a therapeutic target in melanoma and dissected its role in shaping the tumor microenvironment by modulating the fate of tumor, endothelial and immune cells.

## 2. Materials and Methods

### 2.1. Reagents

The following antibodies were obtained from eBioscience (San Diego, CA, USA): anti-CD4 (1/1000; clone 6 K1.5), anti-F4/80 (1/200; clone BM8) and anti-GR-1 (1/500; clone RB6-8C5). Anti-CD8 (1/500; clone 53-6.7), anti-Granzyme B (1/300; clone 6B11), anti-PD-1 (1/300 29F.1A12) and anti-CD206 (1/200; clone C068C2) antibodies were from Biolegend (San Diego, CA, USA). BD Pharmingen provided anti-TNF (1/300; clone MP6-XT22), anti-IFN-γ (1/300; clone XMG1.2) and anti-CD107a (1/1000; clone 1D4B) antibodies. Anti-PCNA (proliferating cell nuclear antigen) was from BioGenex (San Ramon, CA, USA) and anti-VE-Cadherin (clone MAB1002) from R&D Systems (Minneapolis, MN, USA).

### 2.2. Cell Culture

B16.F1 murine melanoma cells were cultured in 100 mm culture plates with Dulbecco’s modified Eagle’s medium supplemented with 5% fetal bovine serum (FBS, Natocor, Córdoba, Argentina). The cultures were maintained at 37 °C in humidified atmosphere at 5% CO_2_.

### 2.3. Mice

WT and TNFR1 KO C57BL/6 mice were housed in the animal facility of IMIBIO-SL and UNSL in temperature-controlled rooms, kept on a 12-h light/dark cycle with unrestricted access to food and water and used as described [21,22]. The genotype of the mice was corroborated by multiplex polymerase chain reaction (PCR) (TNFR1 WT: 5′-TGTGAAAAGGGCACCTTTACGGC-3′, TNFR1 KO 5′-ATTCGCCAATGACAAGACGCTGG-3′, TNFR1 both: 5′-GGCTGCAGTCCACGCACTGG-3′). All experiments involving animal were performed according to national (Law 14346 regarding animal protection) and international (Guide for the Care and Use of Laboratory Animals: Eighth Edition) [23] regulations and were approved by Institutional Committee for Care and Use of Laboratory Animals at UNSL, San Luis, Argentina.

### 2.4. Mouse Melanoma Model

B16.F1 melanoma cells (2.5 × 10^5^ cells) were subcutaneously inoculated into the right dorsal flank of 8–12 weeks-old WT and TNFR1 KO male mice previously anesthetized with Ketamine/Xylazine 90:10 as described [24]. Viability of implanted B16.F1 cells was higher than 95% as assessed by Trypan blue exclusion method. Tumor volume (V) was calculated using a caliper with the formula V = *d*^2^ × *D* × 0.5, where *d* is the smallest and *D* is the largest diameter of each tumor. The day 19th post inoculation animals were sacrificed by inhalation of CO_2_ and tumor masses were removed. After determination of the tumor weight, the tissue was processed for subsequent studies. To study spontaneous metastasis in brain and lungs, 1 × 10^5^ B16.F1 melanoma cells were inoculated and tissues examined after 30 days.

### 2.5. Preparation of Conditioned Medium

Tumor conditioned medium (CM). Fresh (100 mg) tumor tissue was mechanically disrupted in 1 mL of phosphate buffered saline (PBS) 1× by rubbing between frosted slides. The tissue homogenate was collected and centrifuged at 11,000× *g* 10 min at 4 °C. The supernatant (CM) was maintained at −80 °C until use.

Peritoneal macrophages conditioned medium (MoCM). 4% sterile thioglycollate (1 mL) was intraperitoneally inoculated in TNFR1 KO and WT mice. Three days later, the peritoneal exudate was collected and washed twice with sterile physiological saline solution (0.9% *w*/*v* NaCl) by centrifugation at 500× *g*. The pellet was suspended in Dulbecco’s Modified Eagle Medium (DMEM) and cell concentration adjusted to 2 × 10^6^ cells/mL. Subsequently, 4 × 10^6^ cells were seeded in each well of a 6-well plate and cultured with DMEM supplemented with 10% FBS, 50 μM 2-mercaptoethanol and 100 U/L penicillin/streptomycin. After 24 h of incubation, 3 washes were performed with PBS to remove unbound cells. Attached cells were cultured with serum-free DMEM for additional 24 h to generate the MoCM. The MoCM was collected, centrifuged at 500× *g* for 5 min and the supernatant maintained at −80 °C until use.

### 2.6. Cell Migration

Cell migration was performed as described previously [25,26]. Briefly, B16.F1 cells were deprived of fetal bovine serum (FBS) for 18 h. MoCM and CM were added to the lower wells of a modified Boyden Chamber and covered with a 12 μm filter previously coated with 10 μg/mL fibronectin. Cells (50,000) were loaded in the upper wells and permitted to migrate for 6 h at 37 °C in a humidified atmosphere with 5% CO_2_. Non-migrating cells were removed mechanically with a cotton swab. Migrating cells were fixed with methanol and stained with 0.1% Toluidin solution. The membrane was scanned and analyzed with the software Image J. Each data point is the average densitometric data of three individual wells.

### 2.7. Histological Procedure

A representative portion of fresh tumor tissue was separated and fixed in 4% formaldehyde for 24–36 h at room temperature. The tumor pieces were dehydrated with increasing ethanol concentrations, cleared in xylene and embedded in paraffin. Six sections 5 μm thick were obtained using a microtome (Reichert-Jung Hn 40). Two sections were mounted on slides and stained with hematoxylin-eosin (H&E). The remaining four sections were mounted on gelatinized glasses and processed for immunohistochemistry.

### 2.8. Immunohistochemistry

Sections were first deparaffinized with xylene and hydrated by immersion in decreasing concentrations of ethanol. Antigen retrieval was performed by microwaving the sections at maximum power for 3 min (twice) in sodium citrate buffer 0.01M pH 6 and endogenous peroxidase activity was inhibited with 3% H_2_O_2_. After blocking with non-immune serum (1% bovine serum albumin (BSA), 0.09% sodium azide and 0.1% Tween-20) sections were incubated overnight with mouse monoclonal antibody anti-PCNA (ready to use) in humidified chamber at 4 °C. Immunohistochemical visualization was performed using the Super Sensitive Ready-to-Use Immunostaining kit (BioGenex, San Ramon, CA, USA). Briefly, sections were incubated for 30 min with biotinylated anti-IgG followed with 30 min incubation with HRP-conjugated streptavidin. Immunohistochemical reactions were developed using a freshly prepared solution of 3,3′-diaminobenzidine tetrahydrochloride chromogen and H_2_O_2_ substrate. Sections were counterstained with Harris’ hematoxylin, dehydrated and mounted with Entellan. Negative controls were performed by replacing primary antibodies with 10% non-immune serum. No positive structures or cells were found in these sections. The percentage of PCNA^+^ cells was determined using the formula (A/A + B) × 100 [27], where A is the number of immunoreactive cells and B is the number of nuclei in immunonegative cells. The microscopic fields were examined under a 400× magnification.

### 2.9. Indirect Immunofluorescence Analysis

Deparaffinized and rehydrated sections were incubated with anti-VE-Cadherin (1/100) in 5% normal serum 1% BSA, 0.025% Triton X100 for 12 h at 4 °C followed by 1 h incubation at room temperature with Alexa-Fluor-594 anti-rabbit secondary IgG antibodies (1/500, Cell Signaling, Danvers, MA, USA). Microvessel density (MVD) quantification was assessed by counting the number of small vessels (<50 µm) per mm^2^ of tumor. At least 10 fields were counted per tumor and the mean of at least three tumors per experiment was represented. Vessel diameter was calculated by measuring the major diameter of each vessel using ImageJ software. Twenty vessels per tumors were measured and the mean of at least three tumors per experiment represented. Values were normalized to the tumor area assessed at a 200× magnification. All histological determinations were performed by two operators in a double-blind manner and data was pooled.

### 2.10. Quantitative Real-Time PCR

Total RNA was isolated from 30 mg of fresh tumor tissue with RNAzol and reverse transcribed with ProtoScript First Strand cDNA Synthesis kit (New England BioLabs, Ipswich, MA, USA) according to the manufacturer’s instructions. Quantitative real-time PCR was performed with FastStart Universal SYBER Green Master (Roche, Basilea, Suiza) using the following primers: Fw β-act: 5′-TCTACAATGAGCTGCGTGTG-3′, Rv β-act: 5′-ATGGCTGGGGTGTTGAAG-3′, Fw mCCL-2: 5′-AGGTCCCTGTCATGCTTCTGG-3′, Rv mCCL-2: 5′-CTGCTGCTGGTGATCCTCTTG-3′. The cDNAs were diluted 10-fold (for the target genes) and amplified by using the ABI 7500 Fast cycler. Gene expression levels were normalized to β-Actin levels. Quantification was done by the standard CT method.

### 2.11. Measurement of Cellular Melanin Content

Tumor tissue (300 mg) were incubated with 400 μL dissolution buffer (1 M NaOH, 15% DMSO in PBS) for 90 min at 70 °C vortexing every 15 min to solubilize the melanin. After dissolution, the lysate was centrifuged 10 min at 300× *g*. The supernatant was added to a 96-well microplate and the absorbance was read to 475 nm. The data was normalized to protein content.

### 2.12. Gelatin Zymography

To evaluate the tumor gelatinolytic activity, 50 mg of fresh tissue was homogenized in 250 μL of PBS 1X, centrifuged at 11,000× *g* for 15 min at 4 °C and the cell supernatant was separated and stored at −80 °C until use. Equal amount of proteins (70 μg) were subjected to sodium dodecyl sulfate-polyacrylamide gel electrophoresis (SDS-PAGE) under non-reducing conditions. The gels were copolymerized with 1.5% as previously indicated [26]. Two gels were run at once and after SDS removal, both gels were incubated for 42 h at 37 °C with gentle shaking. One of the gels was incubated in developing buffer (50 mM Tris, 5 mM CaCl_2_, 150 mM NaCl, 0.02% NaN_3_) to allow that partially renatured enzymes degrade the gelatin, while the other gel (negative control) was incubated in developing buffer containing 20 mM ethylenediaminetetraacetic acid (EDTA) leading to disappearance of bands produced by metalloproteinases. The degradation was visualized by staining with Coomassie blue. The gel was scanned and analyzed with Image Studio Lite 5.2 software (LI-COR Biosciences, Lincoln, NE, USA).

### 2.13. Measurements of VEGF, IL-10 and IL-12

Levels of vascular endothelial growth factor (VEGF) in mice serum and tumor CM as well as IL-10 and IL-12 in tumor CM were determined using enzyme-linked immunoassay (ELISA) kits (R&D Systems, Minneapolis, MN, USA).

### 2.14. Analysis of Immune Cell Infiltration by Flow Cytometry

A fraction composed mainly of tumor cortex was collected and disaggregated by incubating 60 min at 37 °C in digestion buffer (200 U/mL Collagenase P), 100 U/mL DNase I in DMEM 10% FBS. Cells were washed twice with PBS and centrifuged at 300× *g*. The pellets were incubated with fluorescently-labeled specific antibodies (Zombie Green Fixable Viability Kit, anti-CD45, anti-CD3, anti-CD4, anti-CD8, anti-F4/80, anti-GR1, anti-CD206 or isotype controls) on ice in the dark for 30 min and then washed. The labeled immune cells were resuspended in fluorescence-activated cell sorting (FACS) buffer and fixed for later detection in FACS Aria III (Becton Dickinson, Franklin Lakes, NJ, USA) flow cytometer. TAMs were identified as CD45^+^, Gr1^−^, F4/80^+^ myeloid cells. CD206 was used as a marker of M2-polarized macrophages [28]. T cell infiltration was determined as CD45^+^, CD3^+^ and CD4^+^ or CD8^+^ cells.

### 2.15. Functional Analysis of CD8^+^ T Cell Activation

Splenic CD8^+^ T cells were obtained from C57/BL6 WT or TNFR1KO mice by magnetic purification (Dynabeads Untouched Mouse CD8 cells kit, Invitrogen, Carlsbad, CA, USA)and subsequently seeded (1 × 10^6^/mL) in 10% FBS RPMI (Gibco, Gaithersburg, MD, USA) in a p48 cell culture plate. In vitro stimulation was performed with plate-bound anti-CD3 antibody (2.5 µg/mL; BD Biosciences, San Jose, CA, USA) and anti-CD28 antibody (2 µg/mL; BD Biosciences, San Jose, CA, USA) After 96 h, the cells were harvested, centrifuged at 200× *g*, resuspended in B16 conditioned medium, seeded in a p48 cell culture plate and incubated ON at 37 °C, 5% CO_2_. The Zombie Green Fixable Viability Kit (Biolegend, San Diego, CA, USA) was used to exclude dead cells. Surface proteins were stained with fluorochrome-conjugated antibodies diluted in phosphate-buffered saline 1% Bovine Serum Albumin (PBS 1% BSA) for 20 min at 4 °C. For intracellular staining, cells were permeabilized with BD Perm/Wash buffer (BD Biosciences) and further stained with fluorochrome-conjugated antibodies. To assess cytokine production, CD8^+^ T cells were incubated with PMA (50 ng/mL, Sigma, St. Louis, MO, USA), ionomycin (1 µg/mL, Sigma, St. Louis, MO, USA) and monensin (GolgiSTOP, BD Biosciences, San Jose, CA, USA) at 37 °C. After 4 h, cells were harvested and intracellular cytokines were evaluated by flow cytometry. Samples were acquired in BD Accuri C6 Plus flow cytometer and data was analyzed with FlowJo V10.7.1 software.

### 2.16. Statistical Analyses

Results are expressed as the mean ± SEM of at least three independent experiments. Statistical significance was analyzed by a two-tailed Student’s *t* test or one-way ANOVA followed by Bonferroni post-test as indicated. Differences were considered to be statistically significant when *p* values were <0.05 (* *p* < 0.05; ** *p* < 0.01; *** *p* < 0.001. n.s., not significant).

## 3. Results

### 3.1. Lack of TNFR p55 Signaling Delays Tumor Growth

**Figure 1:**
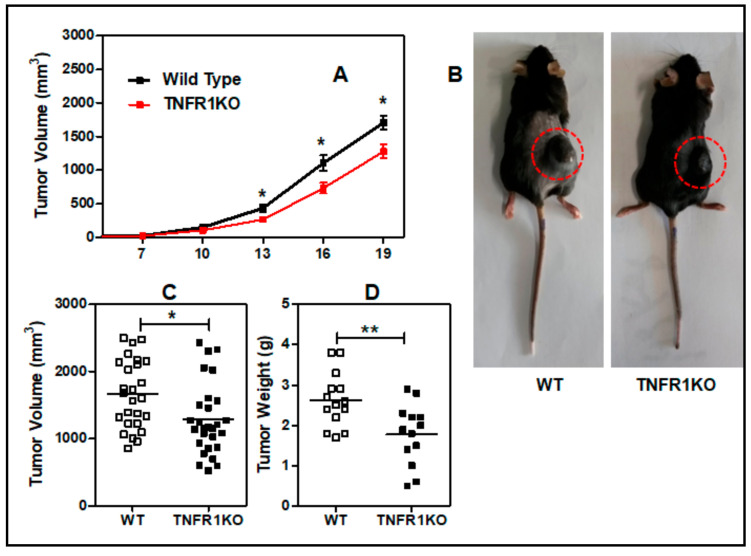
Lack of TNFR1 impairs tumor growth in the B16.F1 melanoma model. (**A**) B16F1 melanoma cells were subcutaneously implanted into WT and TNFR1 KO mice and tumor volume was determined by the formula d^2^ × D × 0.5 at the indicated days. Data are mean ± SEM of 5 independent experiments (5–6 mice per group). (**B**) Representative photographs of WT and TNFR1 KO-bearing mice at day 19 post-inoculation (**C**) Tumor volume (**D**) and weight at day 19 post-inoculation. Values were determined at the indicated days for individual tumors. Bars represent mean values.* *p* < 0.05; ** *p* < 0.01. d: smaller diameter; D: larger diameter.

To evaluate the role of TNFR1 in melanoma, we challenged C57BL/6 WT and TNFR1 KO mice with a subcutaneous injection of B16.F1 mouse melanoma cells. Interestingly, tumor growth was significantly delayed in TNFR1 KO mice starting from day 13 post-inoculation (Figure 1A,B). Likewise, tumor volume and weight (Figure 1C,D) corresponding to day 19 were also significantly lower in TNFR1 KO mice. As assessed by flow cytometry, B16.F1 cells we used did not express TNFR1 (data not shown); thus TNFR1 signaling was completely absent in the experiments performed in TNFR1 KO mice.

**Figure 2:**
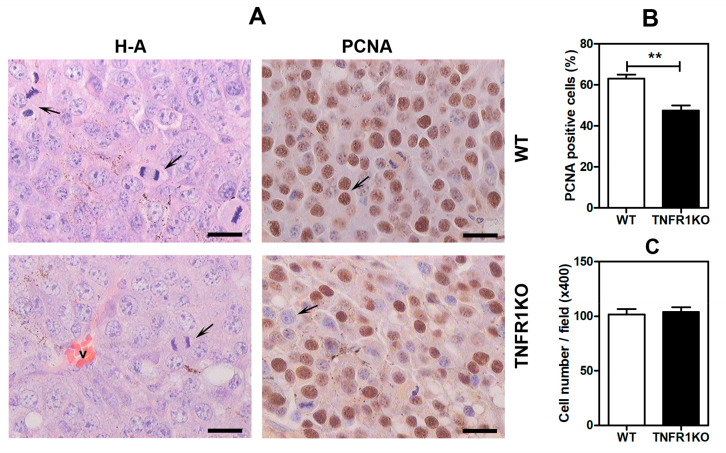
TNFR1 controls tumor proliferation in melanoma. Tumors in WT and TNFR1 KO mice were analyzed by immunohistochemistry to assess expression of proliferating cell nuclear antigen (PCNA), a marker of proliferating cells. (**A**) Hematoxylin & Eosin (H&E) and PCNA staining. The arrows on the H&E panels indicate mitotic cells, the arrows on the right panels indicate PCNA^+^ (top) and PCNA^−^ cells (below). The images are representative of three independent experiments. Scale bar: 25 µm. (**B**) Quantification of PCNA^+^ cells. Data are mean ± SEM (*n* = 3). (**C**) Number of cell nuclei by field. Data are mean ± SEM (*n* = 3), ** *p* < 0.01.

To evaluate the impact of TNFR1 deficiency in tumor cell proliferation we analyzed proliferating cell nuclear antigen (PCNA) in tumor sections. Consistently, tumors developed in TNFR1 KO mice showed significant decrease in PCNA^+^ cells as compared to tumors grown in WT mice (Figure 2A,B). However, we did not observe significant differences in the number of cells per field, indicating a homogeneous cellular distribution and lack of increase in extracellular matrix (ECM) (Figure 2C).

**Figure 3:**
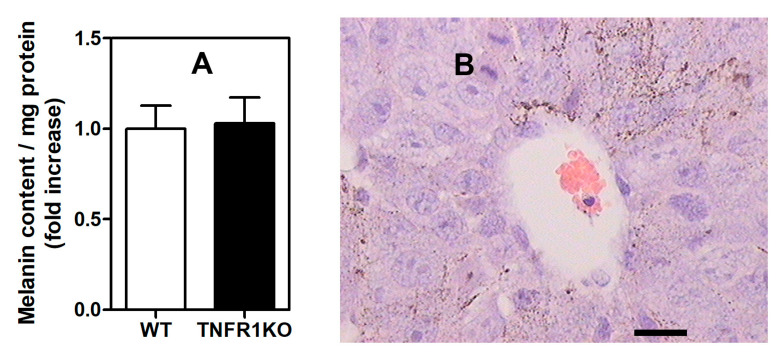
Melanin content is similar in tumors developed in wild type (WT) and TNFR1 KO mice. (**A**) Quantification of cellular melanin normalized to protein concentration. Results are shown as the mean ± SEM of 3 independent experiments and are standardized per mg tumor protein. (**B**) Brown-blackish melanin granules typically observed in hematoxylin-eosin (H&E) stained tumor tissue. Scale bar: 25 µm.

Moreover, the content of melanin—a parameter associated to modulation of melanoma proliferation and migration [29,30]—was similar in both WT and TNFR1 KO tumors (Figure 3).

### 3.2. TNFR1 Deficiency Leads to Impaired Tumor Angiogenesis

**Figure 4:**
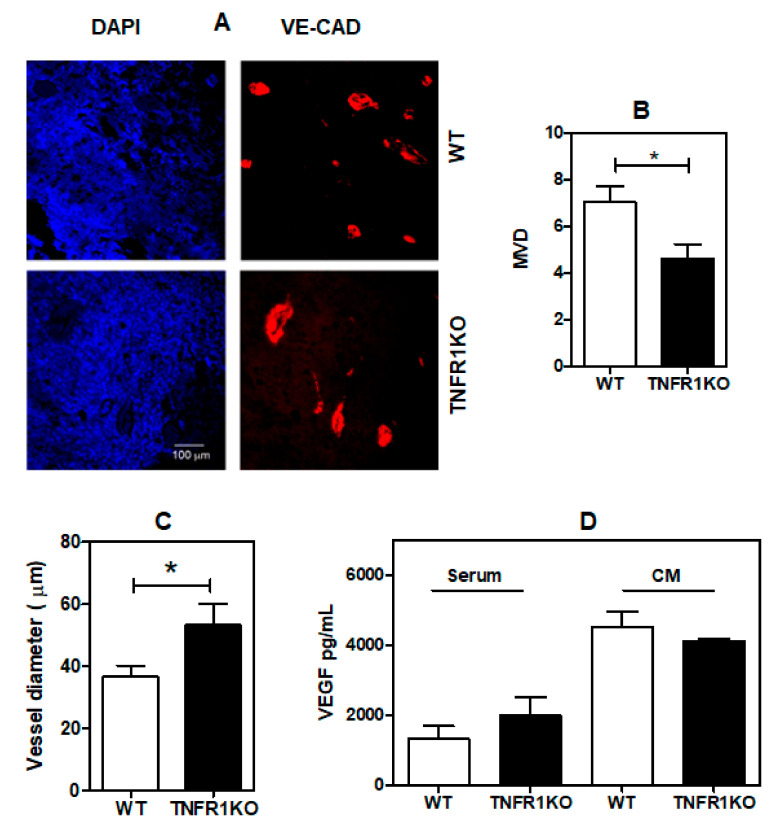
TNFR1 deficiency leads to reduction of tumor-associated vasculature. (**A**) Tumor tissue sections (200× magnification) were processed for immunofluorescence staining. Nuclei were labeled with 4’,6-Diamidino-2-Phenylindole (DAPI) (left) and blood vessels with anti-VE-cadherin antibody (right). (**B**) Relative quantification of blood vessels by specific immunostaining of vascular endothelial (VE)-Cadherin. (**C**) Quantification of the diameter of tumor blood vessels. (**D**) Determination of vascular endothelial growth factor (VEGF) by enzyme-linked immunoassay (ELISA) in serum and CM from WT and TNFR1 KO mice. Results were normalized to total protein concentration. The results are shown as the mean ± SEM of 3 independent experiments analyzed by Student’s *t*-test, * *p* < 0.05.

To further analyze possible mechanisms underlying reduction of tumor growth in TNFR1 KO mice, we evaluated tumor microvasculature by labeling the endothelial antigen VE-cadherin. Accordingly, in tumors implanted in TNFR1 KO mice, angiogenesis was significantly lower and vessels showed higher diameter than that observed in the WT group, suggesting that TNFR1 may control tumor angiogenesis (Figure 4A–C). We then explored whether differences in VEGF levels were associated to changes in blood vessel formation induced by TNFR1 deficiency. We found no differences in systemic and tumor VEGF levels between both groups analyzed (Figure 4D), suggesting that TNFR1 deficiency does not control angiogenesis via indirect regulation of VEGF content.

### 3.3. Impact of Melanoma Conditioned Media from TNFR1 KO Mice in B16.F1 Melanoma Cell Migration

**Figure 5:**
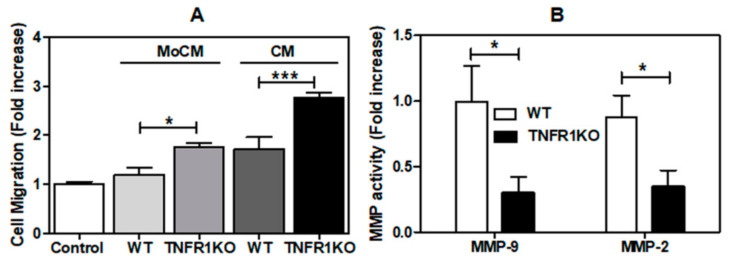
TNFR **c**ontrols migration and invasion of melanoma cells (**A**) Migration of B16.F1 cells induced by peritoneal macrophages CM (MoCM) and tumor CM (CM). The assay was performed in a modified Boyden Chamber (**B**) Zymography assay. Determination of metalloproteinase 2 (MMP-2) and 9 (MMP-9) present in tumor tissue of WT and TNFR1 KO mice obtained at day 19 post-inoculation. The results are shown as the mean ± SEM of 3 independent experiments, * *p* < 0.05, *** *p* < 0.001.

Given that cell migration and invasion are essential processes required for tumor cell metastasis, we evaluated migration of B16.F1 cells exposed to CM from tumor tissue or peritoneal macrophages isolated from WT or TNFR1 KO mice. We found higher migration of B16.F1 melanoma cells exposed to macrophage CM and tumor CM from TNFR1 KO compared to WT mice (Figure 5A). Of note, the activities of MMP-9 and MMP-2, the two major metalloproteinases (MMPs) responsible for ECM remodeling and tumor invasion, were significantly lower in the tumor microenvironment of TNFR1 KO mice (Figure 5B). Although these results may indicate higher metastatic capacity of B16.F1 TNFR1-sufficient tumor cells, we could find no signs of macroscopic spontaneous metastasis in brain and lungs, even after 30 days of subcutaneous inoculation of B16.F1 in WT and TNFR1 KO mice (data not shown).

### 3.4. Tumor-Bearing Mice Lacking TNFR1 KO Display Higher CD8^+^ T Cell Infiltration

**Figure 6:**
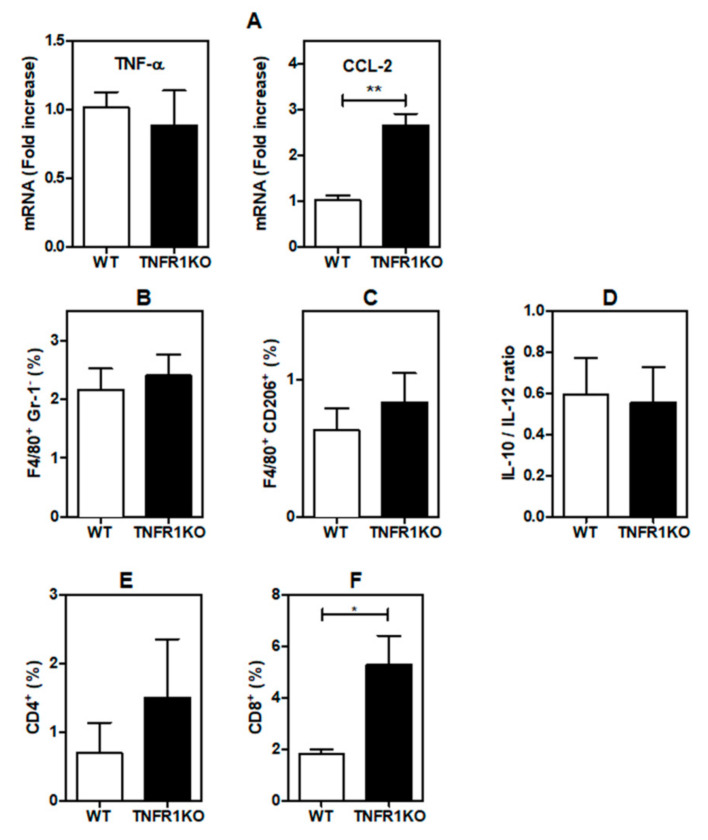
TNFR1 differentially controls immune cell infiltrates in the tumor microenvironment.TNFR1 KO and WT animals were inoculated with B16.F1 cells and analyzed for: (**A**) Expression of TNF-α and CCL-2 in tumor tissue. (**B**) Percentage of total TAMs. (**C**) Percentage of M2-polarized TAMs. (**E**) Frequency of CD4^+^ T cells and (**F**) CD8^+^ T cells in tumor tissue by flow cytometry. (**D**) Ratio of IL-10 and IL-12 concentrations by ELISA. Samples were obtained and analyzed at day 19 post-inoculation. The results are shown as the mean ± SEM of 3 independent experiments, * *p* < 0.05, ** *p* < 0.01.

Finally, we examined whether lack of TNFR1 signaling in the tumor microenvironment may affect the number and/or phenotype of tumor-infiltrating cells. We first analyzed the expression of TNF-α, the TNFR1 ligand and CCL-2, a chemokine involved in monocytes/macrophage recruitment to tumor sites. While we did not find differences in TNF-α expression, levels of CCL-2 were dramatically higher in tumors grown in TNFR1 KO vs. WT tumors mice (Figure 6A). In spite of the increase in CCL-2, the number of total TAMs (F4/80^+^/GR-1^−^) infiltrating tumors was not affected in the absence of TNFR1 signaling (Figure 6B). The phenotype of TAMs is central to define the growth and metastatic capacity of neoplastic cells. Remarkably, we found no differences in TAM displaying M2 phenotype (F4/80^+^/CD206^+^) in tumors developed in WT or TNFR1 KO mice (Figure 6C). In agreement, the IL-10/IL-12 ratio, a critical parameter used to evaluate tolerogenic and immunoregulatory phenotypes [31], was not altered in tumor CM (Figure 6D).

Although both CD4^+^ (Figure 6E) and CD8^+^ (Figure 6F) T cell infiltrates were observed in tumors developed in TNFR1-deficient mice, only CD8^+^ T cells were significantly increased in the stroma of B16 tumor-bearing TNFR1 KO mice (Figure 6F). Thus, TNFR1 signaling may control tumor growth in melanoma by shaping tumor-associated CD8^+^ T cell infiltration.

**Figure 7:**
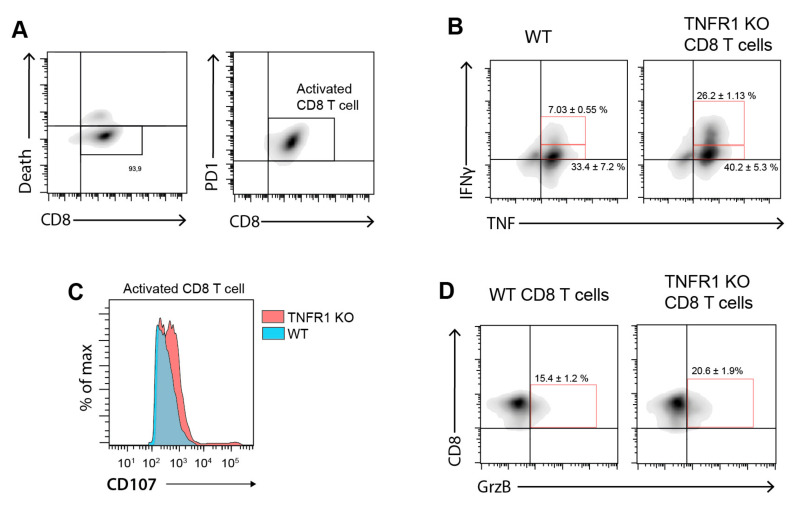
TNFR1 expression modulates activation state of CD8^+^ T cells in the presence of melanoma conditioned media. (**A**) Gating strategy of magnetic-isolated splenic CD8^+^ T cells (left panel) from C57BL/6 WT or TNFR1 KO mice. The right panel shows anti-CD3/CD28-activated CD8^+^ T cells during 96 h in the presence of CM from B16.F1 melanoma cells. (**B**) Intracellular IFN-γ and TNF-α in activated CD8^+^ T cells from WT or TNFR1 KO mice cultured in the presence of CM from B16.F1 cells. (**C**) Degranulation (CD107a^+^) of WT or TNFR1 KO activated CD8^+^ T cells in the presence of CM from B16.F1 cells. (**D**) Granzyme-B production on WT or TNFR1 KO activated CD8^+^ T cells in the presence of CM from B16.F1 cells. DotPlots (**A**,**B**,**D**) and histograms (**C**) are representative plots of at least two independent experiments.

To provide a possible mechanistic explanation for the reduced tumor growth in TNFR1 KO mice and given the increased frequency of CD8^+^ infiltrating T cells, we explored the ex vivo functionality of TNFR1 KO vs. WT activated CD8^+^ T cells cultured in the presence of B16.F1 melanoma cells conditioned media (B16.F1 CM). Isolated CD8^+^ T cells from TNFR1 KO or WT mice were activated during 96 h with anti-CD3/-CD28 antibodies and exposed ON to B16.F1 CM (Figure 7A). Interestingly, CD8^+^ T cells obtained from TNFR1 KO mice showed increased production of pro-inflammatory cytokines TNF-α and IFN-γ compared to WT CD8^+^ T cells (Figure 7B). In addition, TNFR1 KO CD8^+^ T cells exhibited higher cytotoxic potential, evaluated by expression of Granzyme B (GrzB) (Figure 7D) and CD107a mobilization (Figure 7C), as a cell surface marker of degranulation. Therefore, TNFR1 KO CD8^+^ T cells displayed higher activation and increased cytotoxic activity as compared to their WT counterparts.

## 4. Discussion

TNF-α is a versatile cytokine that plays diverse roles in cancer through the engagement of two membrane receptors named p55/TNFR1 and p75/TNFR2 [2,3,7,8,9]. A number of paradoxical effects have been reported indicating that TNF-α may act either as a suppressor or as a stimulator of cancer progression. Here, we show that TNFR1 deficiency results in reduced tumor progression and lower blood vessel formation. Moreover, loss of this cytokine receptor alters the composition of the immune infiltrate in tumor parenchyma favoring the influx and activation of CD8^+^ T cells. Thus, in the context of melanoma, TNFR1 seems to exert a predominant pro-tumoral function.

The role of proinflammatory cytokines in tumor progression has been widely studied. Our results indicate that B16.F1 melanoma cells injected subcutaneously in TNFR1-deficient mice generate smaller local tumors, compared with injections in WT mice. Although recent reports have established that tumor growth was impaired in TNF KO and TNFR1 KO but not TNFR2 KO, those mice were injected with B16K1 melanoma cells, a genetically-modified cell line obtained from B16F10 cells, which stably express the MHC-I (major histocompatibility complex) molecule H-2Kb [15]. Notably, the authors did not find differences in tumor growth when they injected B16F10 or B16-BL6 cells (both of which express low MHC-I) in WT and TNFR1 KO mice, suggesting that expression of MHC-I at the surface of melanoma is important for growth inhibition induced by the lack of TNFR1 in the host [15]. Intriguingly, our data using B16.F1 melanoma cells, which display low MHC-I expression [32], show that tumor growth is reduced in TNFR1 KO mice, suggesting that other MHC-I-independent factors may contribute to the anti-tumoral effect. In addition, although Bertrand et al. showed that TNFR1 is more critical than TNFR2 in reducing B16K1 tumor growth [15], it has also been demonstrated that TNFR2 KO mice also develop smaller tumors after implantation of Lewis Lung carcinoma and B16 cells [33]. Because in the latter report implantation of B16 cells was performed using Matrigel, whether or not these discrepancies could be related to differences in the experimental setting or between cell line variants (B16 vs. B16K1) used are uncertain. Indeed, although it was shown that both B16F10 and B16K1 cells expressed TNFR1 [15], B16.F1 melanoma cells available in our laboratory do not express this receptor. Moreover, recent reports demonstrated that different clones of B16.F1 cells exhibit differences in tumor growth, cytokine secretion and associated immune infiltrates [34].

It is clear, however, that TNFR1 has a complex and sometimes contradictory role in cancer. Certainly, TNFR1 KO mice show more severe colitis-driven carcinogenesis compared to WT animals [13], which is probably related to enhanced inflammatory response. Moreover, pancreatic tumors are larger and more vascularized in TNFR1 KO than in WT mice [35]. Intriguingly, treatment of WT mice with human TNF-α, that only binds and activates murine TNFR1, increases pancreatic tumor growth [35]. Therefore, engagement of TNFR1 may lead to either increased or decreased tumor growth. The final outcome may be dependent not only on the type of tumor but also on time frame of this modulatory effect and the availability and presentation of its canonical ligand TNF-α.

Cell migration is a dynamic process dependent on the presence of a chemoattractant gradient implying cytoskeletal reorganization and formation of structures that enable cell movement [26]. Despite having similar requirements than migration, invasion, one of the hallmarks of cancer, also involves proteolytic degradation of ECM by MMPs, allowing cancer cells to spread out of the primary tumor [36]. Interestingly, aberrant angiogenesis, other hallmark of cancer, is induced early during the development of invasive cancers [36]. Previous findings reported suppression of experimental lung metastases in TNFR1 KO mice injected in the tail vein with murine renal carcinoma cells [37]. Although we could not detect spontaneous brain and lung metastases in our model, our results show that activity of MMP-2 and MMP-9 and angiogenesis were decreased in tumors developed in TNFR1 KO mice, suggestive of a pro-tumoral role of TNFR1 in melanoma progression. On the contrary, CM obtained from TNFR1 KO tumors was more effective in inducing the migration of B16.F1 cells than WT CM, suggesting that TNFR1 may have an anti-tumoral effect. This apparent discrepancy could be due the artificial chemoattractant gradient in the Boyden Chamber used to evaluate migration which could not recapitulate that fond in the tumor microenvironment. Alternatively, it is important to consider that distribution of cells and therefore also the production of endogenous factors is not homogenous in the tumor. Thus, although tumors developed in TNFR1KO mice may produce larger amounts of chemoattractants and downregulation of MMPs activity, the location in the tumor and the source of those factors could be different. Nevertheless, it is tempting to speculate that TNFR1 may play a dual role following a particular timing during melanoma progression: at early stages it may support melanoma proliferation while reducing migration whereas at later stages it could foster activity of metalloproteinases that might eventually lead to metastasis. Although we could find no significant decrease in the metastatic potential of B16.F1 cells in TNFR1 KO mice, this could be due to the augmented migratory capacity of B16.F1 observed when exposed to TNFR1 KO mice CM. Moreover, since B16.F1 melanoma cells show low metastatic capability, whether the final outcome of these opposite effects in migration and MMP-2 and MMP-9 activities would be pro- or anti-metastatic needs to be addressed. In that regard, further studies involving experimental metastatic models or more invasive B16F10 cell lines could help elucidate the role of TNF-α in the metastatic cascade.

The relationship between melanin content and proliferation of melanoma cells is controversial. Melanin is formed by enzymatic reactions in which L-tyrosine is converted to heterogeneous melanin in melanocytes. Many reports highlight the role of melanin in diverse aspects of melanoma biology, including cell death [38], chemo- and radiotherapy [18], lymphocyte viability, toxicity and production of proinflammatory cytokines [39]. Remarkably, the pigmented phenotype correlates with poor prognosis in metastatic melanoma patients [18,19] and metastasis in uveal melanoma [40], suggesting that tumor melanin content is a negative prognostic factor in melanoma patients. While some reports indicate that an increase in melanin content is associated with a decreased proliferation of B16 melanoma cells [41], others have shown that an increase in melanin leads to enhanced B16 cell proliferation both in vitro and in vivo [29]. Moreover, a decrease in melanin has also been reported to reduce B16 cell migration [30]. Thus, we decided to explore whether a distinct tumor microenvironment in WT and TNFR1 KO mice could affect melanin content of the tumors generated in those mice. However, in our model, we could find no differences between the levels of melanin in WT and TNFR1 KO tumors. On the other hand, TNFR1 KO tumors displayed reduced PCNA expression, suggestive of a decrease in cell proliferation of melanoma cells. Since the cell size was similar in both tumors, it is likely that the reduction in tumor growth in TNFR1 KO mice correlates, at least in part, with decreased cell proliferation.

Although Bertrand and colleagues found considerable differences in T-cell trafficking and content of high endothelial venules, they did not explore the role of TNFR1 in neovascularization [15]. Since development of new vessels is required to fulfill the increased demand of nutrients and oxygen by tumor cells [36], we sought to explore the importance of this process in our model. Despite the fact that VEGF levels are similar in WT and TNFR1 KO mice, we found that expression of VE-cadherin, a typical endothelial cell marker, was considerably lower in tumors developed in TNFR1 KO mice, suggesting that a decrease in microvessel density may account for the reduction in tumor growth observed in those mice. Accordingly, a comparable effect was previously reported in a similar model with B16 melanoma cells, showing that tumor growth and angiogenesis was reduced in IL-1β KO mice as compared to WT mice [42]. Likewise, in a model of oxygen-induced retinopathy that resembles the hypoxic tumor microenvironment, TNFR1 KO mice exhibited a decrease in neovascularization [43]. Moreover, it was previously shown that the vascular density in the lung metastatic tissue was less apparent in TNFR1 KO than in WT mice injected with murine renal carcinoma cells [37]. Thus, our data suggest that reduced tumor progression in TNFR1 KO mice is probably caused, at least partially, by a decrease in angiogenesis. In contrast, it has been shown that TNFR1 KO mice display larger and highly vascularized pancreatic tumors [35] as well as increased angiogenesis and healing when wounds were induced in the skin [44]. Hence, the role of TNFR1 in angiogenesis is likely dependent on the type of tumor or underlying inflammatory disease. While activation of this pathway may offer benefits to some pathophysiologic processes including wound healing, it may be detrimental for development of anti-tumor responses.

The tumor microenvironment (TME) allows tumors to express their full neoplastic phenotype and is composed by various cell types, including endothelial cells, TAMs, cancer-associated fibroblasts (CAFs), neutrophils, mast cells, lymphocytes and myeloid-derived suppressor cells (MDSCs) among others. This prompted us to analyze the nature and composition of tumor-immune contexture in WT and TNFR1 KO mice. First, we found that expression of CCL-2 was significantly higher in TNFR1 deficient mice, suggesting that tumors developed in those mice exhibit a pronounced infiltration of TAMs, which display a permissive M2-like phenotype, thus promoting angiogenesis, immunosuppression and cancer progression [45]. Surprisingly, we found no differences in the infiltrate not only of M2-polarized TAMs but also of total TAMs. In agreement, the IL-10/IL-12 ratio, a critical parameter used to distinguish the macrophage polarization profile [31], was similar in both WT and TNFR1 KO mice.

Recent reports indicate that tumors developed in TNF-α–deficient mice displayed enhanced infiltration of CD8^+^ T lymphocytes [15]. Also, tumors generated by implantation of B16K1 melanoma cells in TNFR1 KO mice, revealed greater accumulation of CD8^+^ T cells in tumor parenchyma [15]. Moreover, it has recently been shown that regression of breast cancer growth in IL-1β KO mice is related, at least in part, to an increase of activated CD8^+^T lymphocytes [46]. Our findings are in line with those results, showing that infiltration of CD8^+^ T cells was considerably higher in tumors developed in TNFR1 KO mice. Accordingly, functional ex vivo experiments demonstrated that TNFR1 KO CD8^+^ T cells display higher cytotoxic potential as assessed by increased GrzB, TNF-α, IFN-γ and CD107a expression. Although the ratio of CD8^+^/CD4^+^ T cells was higher in TNF KO as compared to WT mice [15], we found no significant differences in WT vs. TNFR1 KO mice. Considering that development of CD4^+^ and CD8^+^ T cells is similar in WT, TNFR1 KO and TNFR2 KO [47], it is tempting to speculate that TNF-α/TNFR1 signaling may control lymphocyte migration toward sites of injury. Accordingly, lesions generated in *Leishmania major*-infected TNFR1 KO mice are persistent and display an increased infiltrate of CD8^+^ T cells [48]. Although other report demonstrated that TNFR1 KO mice are more susceptible to infection with *Listeria monocytogenes* [47], the role of CD8^+^ T cells in this process has not been examined. Therefore, TNFR1 seems to control chronic inflammation by inhibiting not only the accumulation but also the cytotoxic potential of CD8^+^ T cells.

## 5. Limitations

This study has some experimental limitations. First, the model of subcutaneous inoculation of B16.F1 cells does not completely recapitulate human disease. Thus, it will be important to further extend these studies to other models in the future. Second, the studies regarding migration, invasion and analysis of immune cell infiltration were performed by using a representative portion of fresh tumor tissue. Consequently, we cannot be fully certain that spatial distribution of immune cells changes in TNFR1 KO vs. WT mice. Indeed, a difference in location of immune cells may also account for different local production of endogenous factors that may affect migration, invasion and angiogenesis. Moreover, although we detected an increase in CD8^+^ T cells in the tumors of TNFR1 KO mice, it remains an open question whether this T cell subpopulation interacts with melanoma cells.

## 6. Conclusions

In summary, our findings indicate that, in melanoma settings, TNFR1 signaling has a pro-tumoral function. This tumor-promoting activity may be, at least in part associated with control of angiogenesis and CD8^+^-dependent T-cell activation and influx to the TME. Thus, targeting TNFR1 signaling, using selective antagonists, may contribute to reduce melanoma cell growth via immune-dependent or -independent mechanisms.
